# Royal Society of Medicine

**Published:** 1913-05-31

**Authors:** 


### Royal Society of Medicine.

### ANTHRAX IN THE WOOL INDUSTRY.

At the meeting on Friday, May 23, of the Section of Epidemiology and State Medicine, Dr. Hamer in the chair, Dr. F. W. Eurich read a paper on "Anthrax in the Woollen Industry, with Special Reference to Bradford." During the last half-century there occurred among the operatives in this industry forms of bronchitis, pneumonia, and so-called blood-poisoning of a peculiarly deadly nature. A notable landmark in the history was when a conference of representatives of employers, workpeople, health committees, and the medical profession took place, and certain regulations, known as "The Bradford Rules," were drawn up. These were voluntarily carried into effect, and formed the basis of the subsequent Home Office Regulations. These precautionary measures greatly reduced the number of cases, especially of the pulmonary variety, and particularly among people who had to handle the raw material before it was washed. But the constant cropping up of cases showed that some important causative factor had not received due attention, and the recognition of this fact led to the formation of the "Anthrax Investigation Board."

Dr. Eurich then, by the aid of some excellent photographs, described the various processes in the manufacture. In these a great deal of dust is thrown out, in which anthrax bacilli are found. Much is done by arranging for part of the process to be carried out on wire netting, beneath which a down draught is produced, so as to draw away the dust from the worker. Dr. Eurich has full records of 110 cases. Of these, nineteen were of the internal variety, and they were all fatal; thirteen only of the remaining ninety-one external cases were fatal. The commonest situations of the pustules were the neck (28), the cheek (13), and the forearm (10). Of the twenty-eight neck cases, seven died. He emphasised the very great importance of absolute rest to the affected part in the treatment. The opsonic index is low during the active stages of the disease, and rises to the normal, or even higher, after the pustule has been excised, or when spontaneous recovery has ensued.

Microscopic examination of serum and blood does not alone suffice for diagnosis: cultural methods should be employed in all cases, though even then failure to cultivate the bacillus does not preclude the possibility of it being anthrax. Bradford surgeons practise excision of the pustule whenever possible, and in most cases also Sclavo's serum is injected—80 c.c. intravenously, followed in twenty-four hours by 60 c.c. Should this be refused by a patient vaccine is to be recommended.

An analysis of the nineteen fatal internal cases is instructive. Four had no medical attention; eight were already moribund when skilled advice was sought; in the remaining seven the nature of the illness was not suspected until the patient was collapsed; in fact, in two it was the unexpected sudden death of the patient which first aroused suspicion.

